# Of Masks and Men? Gender, Sex, and Protective Measures during COVID-19

**DOI:** 10.1017/S1743923X20000616

**Published:** 2020-12

**Authors:** Dan Cassino, Yasemin Besen-Cassino

**Affiliations:** 1 Fairleigh Dickinson University; 2 Montclair State University

**Keywords:** Masculinity, COVID-19, gender identity, men's health

## Abstract

Since the beginning of the COVID-19 pandemic in the United States, men have been consistently less likely to report wearing a protective face mask. There are several possible reasons for this difference, including partisanship and gender identity. Using a national live-caller telephone survey that measures gender identity, we show that men's gender identities are strongly related to their views of mask wearing, especially when gender identity is highly salient to the individual. The effects of this interaction of sex and gender are shown to be separate from the effects of partisanship. While partisanship is a significant driver of attitudes about face masks, within partisan groups, men who report “completely” masculine gender identities are very different from their fellow partisans.

According to recent guidance from public health experts (WHO 2020), wearing a mask is one of the most important things people can do to slow the spread of COVID-19. Yet people in the United States are not wearing them as much as experts recommend.

Data from the Understanding America Study (UAS) panel survey carried out by the University of Southern California's Dornsife Center for Economic and Social Research shows that while mask use increased dramatically over the first months of the pandemic, U.S. men have been significantly less likely than U.S. women to report having worn face masks throughout, as Figure 1 shows.

**Figure 1.**
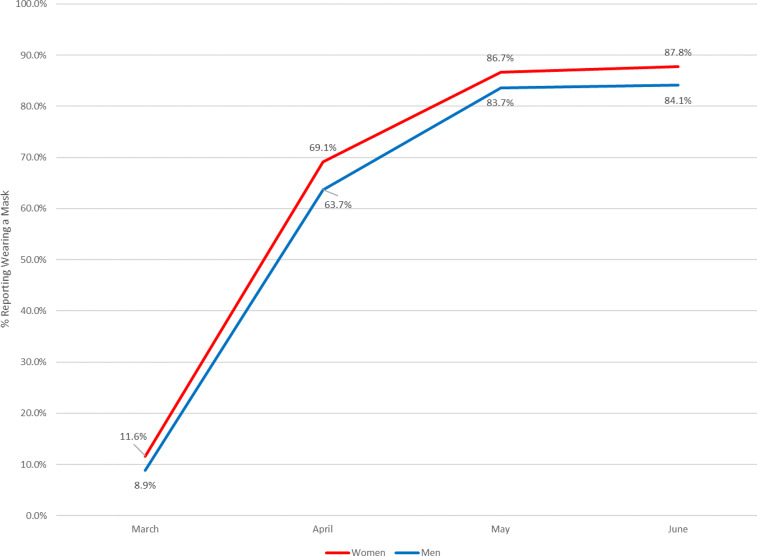
Reported mask wearing by sex. Author's calculation.

In the same data, Democrats are more likely than Republicans to report having worn a mask, but there are no significant differences between men and women within partisan groups. It makes sense that partisanship would impact mask-wearing behavior, as leaders within the Republican Party have consistently signaled that the virus is not a serious threat and that mask wearing is not important.

So what is going on? Are men disproportionately failing to wear (or at least report wearing) face masks because they are more likely to identify as Republicans or because of gender? While extant data mostly looks at sex, few scholars think that such differences are driven by biology. As Schneider and Bos (2019, 175) put it, “Sex differences . . . require gendered explanations.” In other words, an observed difference between men and women is likely a difference between masculine and feminine identities that is not being measured.

To account for the roles of both gender and partisanship in mask-wearing attitudes, we make use of a national live-caller telephone survey carried out by the FDU (Fairleigh Dickinson University) Poll in late May 2020 with an overall sample size of 1,003. In it, we ask respondents about their gender identity as well as the importance of this gender identity to them.

Research on masculinity and health outcomes has focused on the extent to which masculinity leads men to avoid prophylactic health measures and to be less likely to seek treatment for health problems that do arise. In the United States, men's life expectancy is seven years shorter than women's, men have higher death rates from the top 15 causes, and they are more likely to suffer from chronic conditions (Courtenay 2000).

These health differences have been explained using gender theory (Courtenay 2000). Borrowing from Connell's (1995) hegemonic masculinity, Courtenay shows that men's performance of gender prevents them from seeking health care and following health guidelines. For example, they are less likely to use preventive medicine (Courtenay 2000; Gibson and Denner 2000), seek medical help, or adopt a healthy lifestyle (Mahalik, Burns, and Syzdek 2007). Research on masculinity (Sabo 2013) has shown that this is driven in part by a desire to be seen as a Superman: men see invincibility as a central component of masculine identity, and to seek medical help would be to admit vulnerability.

Such studies are directly relevant to mask wearing and other behaviors intended to limit the spread of COVID-19. To the extent that men are refusing to wear masks or opposing mask requirements, we expect these attitudes are correlated with both masculinity and the importance of gender to the respondent's overall identity.

## DATA

**Figure 2.**
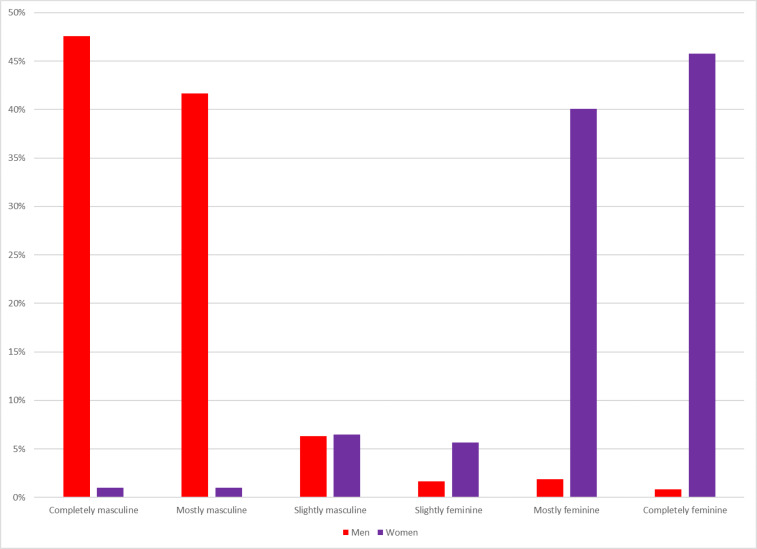
Reported gender identity by sex. Author's calculation.

The FDU Poll includes a number of measures designed to get at the relationship between gender identity and responses to COVID-19. For instance, the respondent's sex was asked within the survey, rather than being guessed by the interviewer; 51% were women. Respondents were asked about their gender identity using a 6-point unidimensional scale running from “completely masculine” to “completely feminine.” Among the respondents, 48% of men and 46% of women identified as “completely” masculine or feminine, with most of the remaining identifying as “mostly” in the gender category in line with their sex; see Figure 2.

Of course, this is far from the only way to measure gender identity. The most comprehensive approach is the Bem Sex Role Inventory (BSRI; Bem 1981), which has been used in political surveys by McDermott (2016). However, the length of the BSRI has led to the development of other approaches, such as the 101-point unidimensional scaled used by Bittner and Goodyear-Grant (2017b) or Westbrook and Saperstein's (2015) multidimensional scales. Multidimensional scales like those used by McDermott (2016) or Westbrook and Saperstein (2015) better align with our theoretical understanding of gender than unidimensional scales, but in U.S. general population samples using self-reported scales, the dimensions of masculinity and femininity are sufficiently negatively correlated as to make multi-item scales inefficient from a survey design perspective (Cassino and Besen-Cassino 2020). They are less correlated in scales like the BSRI, but the results from the two types of scales, in U.S. general population surveys, are broadly similar. While using a single item to ask respondents to describe their gender identity is far from perfect, it has the advantage of being compact, generally aligns with other gender identity measures, and is far preferable to using sex as a proxy for gender identity.

Respondents were also asked how important being a man or a woman is to their overall identity, using a 4-point scale running from “extremely important” to “not important at all.” Among the respondents, 43% of men and 55% of women said that it was “extremely important,” with most of the remainder in the next category, “somewhat important.” This measure was inspired by Bittner and Goodyear-Grant's work (2017a) showing the differential impact of gender identity by the reported importance of that identity to the individual.

## GENDER, PARTISANSHIP, AND COVID-19 RESPONSES

Respondents were asked a series of questions about policies designed to limit the spread of COVID-19, including mask requirements. As Table 1 demonstrates, on four of these items, men report significantly lower support than women. However, the figures also make it clear that the gap between men and women is less about sex than about gender. Among men, 56% say that a ban on large public gatherings is acceptable; among men who say that they are “completely masculine,” that figure drops to 48%. Among men who place themselves anywhere else on the gender identity item, support for a ban on large gatherings is 64%. As such, it would be perfectly reasonable to divide up the population not based on sex but between men who identify as “completely” masculine, who have low support for these COVID-reduction measures, and everyone else, who have higher, but generally not distinguishable from each other, support for the measures.

**Table 1.** Percentage supporting COVID-19 measures, by sex and gender

	Male, Completely Masculine	Male, not Completely Masculine	Female, Completely Feminine	Female, Not Completely Feminine	All Men	All Women	All Masculine	All Feminine

***Which of the following do you think is acceptable for preventing the spread of coronavirus?***								
*Requiring people to wear face masks while in enclosed public spaces*	**0.56** (**0.07)**	0.81 (0.05)	0.88 (0.04)	0.87 (0.04)	**0.69** (**0.05)**	0.88 (0.03)	0.69 (0.04)	0.88 (0.03)
*Public bans on gatherings of more than 10 people*	**0.48** (**0.07)**	0.64 (0.06)	0.64 (0.07)	0.77 (0.06)	**0.56** (**0.04)**	0.7 (0.04)	0.58 (0.05)	0.7 (0.05)
*Having your temperature checked before entering an enclosed public place*	0.80 (0.05)	0.86 (0.05)	0.86 (0.05)	0.89 (0.04)	0.83 (0.03)	0.87 (0.03)	0.83 (0.04)	0.88 (0.03)
*Mandatory isolation of those who have tested positive for coronavirus*	**0.78** (**0.03)**	0.92 (0.04)	0.89 (0.04)	0.90 (0.04)	0.85 (0.04)	0.89 (0.03)	0.84 (0.03)	0.9 (0.03)
*Mandatory vaccinations by employers and schools against the coronavirus once it becomes available, with limited exceptions for medical and religious reasons*	0.51 (0.06)	0.7 (0.06)	0.6 (0.06)	0.66 (0.07)	0.61 (0.05)	0.63 (0.05)	0.61 (0.05)	0.63 (0.04)
*Allowing the government to alert those whom youve been around if you become infected with coronavirus*	**0.54** (**0.07)**	0.81 (0.05)	0.78 (0.06)	0.79 (0.06)	**0.68** (**0.05)**	0.78 (0.04)	0.68 (0.05)	0.79 (0.04)
*Mandatory sharing of personal health information with the government in order to track the spread of corona virus*	**0.39 (0.06)**	0.6 (0.07)	0.62 (0.07)	0.66 (0.06)	**0.5 (0.05)**	0.64 (0.05)	0.51 (0.05)	0.64 (0.05)

*Notes*: 95% MOEs in parentheses; significant differences in boldface.

Differences between “completely masculine” men and everyone else extends to other COVID-19 related items in the survey as well. Men with gender identities that “completely” conform to their sex are more likely than other groups to think that the number of infections and deaths related to COVID-19 have been exaggerated, for instance.

In some instances, men and women who report the most traditional gender identities (those conforming “completely” to their sex) have different views than everyone else. For instance, both men and women with “completely” conforming gender identities are significantly less likely to say that they would get a coronavirus vaccine (55%) if one were available compared with other Americans (68%).

**Figure 3.**
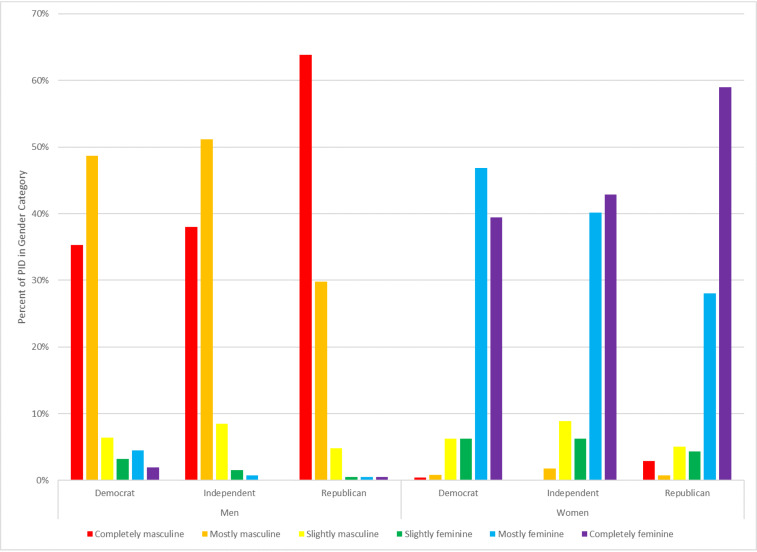
Reported gender identity by sex and partisanship. Author's calculation.

However, Figure 3 suggests that it is still not clear whether these differences are driven by gender identity or by partisanship. Men who identify as “completely masculine” are different from other men on these issues, but they are also more likely to identify as Republican than other men. Among Republican men in the sample, 64% identify as “completely masculine,” compared with only 35% of Democratic men. There are similar, though smaller, differences among women, with Republican women being more likely to identify as “completely feminine” than others.

## ANALYSES

As Republicans are less likely to support government efforts to contain COVID-19, it seems possible that we are conflating effects of gender identity with effects of partisanship, as these identities are closely linked (Cassino and Besen-Cassino 2020). Fortunately, we can disentangle them in this case through regression. We use logit regression, with standard controls included, to model the effects of these factors on support for mask requirements. We present the results of three separate logit models: one with gender identity, the importance of gender identity, and sex interacted; one without any interactions; and one without the importance of gender identity included (either as main effect or in the interactions).

What is evident from these models is the importance of the three-way interaction between sex, gender identity, and the importance of that gender identity. As is clear from Model 2, gender identity matters on its own, but gender importance and sex do not. It is only when we include all three of them, interacted, in the model that we see the full scope of the effect. Substantively, this three-way interaction indicates that gender identity has a larger impact on the attitudes of men than on women, and that this effect is further magnified among those men who say that their gender is very important to their identity.

**Table 2.** Logit regression models for support of mask requirements

	Model 1 (Full)	Model 2 (No interactions)	Model 3 (No Gender Importance)
	Coef	Std Error	Z	Coef	Std Error	Z	Coef	Std Error	Z

Party ID: Independent	**−1**.**85**	**0**.**30**	**−6**.**2**	**−1**.**85**	**0**.**30**	**−6**.**2**	**−1**.**85**	**0**.**30**	**−6**.**3**
Party ID: Republican	**−2**.**03**	**0**.**29**	**−7**.**0**	**−2**.**14**	**0**.**28**	**−7**.**5**	**−2**.**11**	**0**.**28**	**−7**.**4**
Gender Identity	**1**.**27**	**0**.**41**	**3**.**1**	**0**.**25**	**0**.**10**	**2**.**5**	**0**.**51**	**0**.**18**	**2**.**9**
Sex	**5**.**31**	**1**.**95**	**2**.**7**	−0.22	0.40	−0.6	0.68	0.73	0.9
Gender Importance	**0**.**82**	**0**.**28**	**2**.**9**	0.02	0.10	0.2			
Education	0.14	0.09	1.6	0.15	0.09	1.7	0.15	0.09	1.7
Non-White	**0**.**86**	**0**.**28**	**3**.**1**	**0**.**79**	**0**.**27**	**2**.**9**	**0**.**82**	**0**.**27**	**3**.**0**
Age	**0**.**19**	**0**.**09**	**2**.**0**	0.16	0.09	1.8	0.21	0.09	2.2
Constant	−1.58	0.87	−1.8	0.69	0.70	1.0	−0.05	0.64	−0.1
*Interactions*									
Sex x Gender Identity	**−1**.**60**	**0**.**53**	**−3**.**0**				−0.36	0.22	−1.6
Sex x Gender Importance	**−2**.**11**	**0**.**84**	**−2**.**5**						
Gender Identity x Importance	**−0**.**39**	**0**.**15**	**−2**.**6**						
Sex x Gender Identity x Importance	**0**.**57**	**0**.**22**	**2**.**6**						

*Note*: Sample sizes for all of these models is 934, with a pseudo-*r*^2^ for the full model of 0.17.

**Figure 4.**
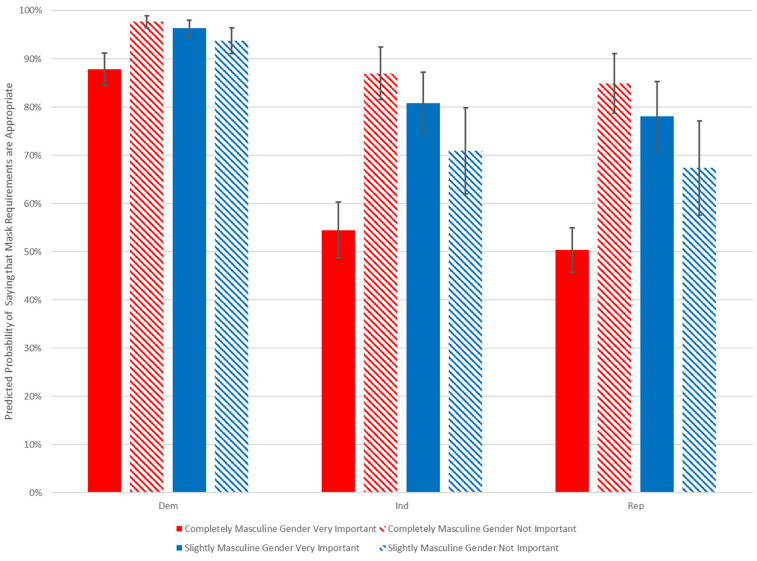
Predicted probabilities of supporting mask requirement, by gender identity, gender importance, and partisanship for men. Author's calculation.

In addition, while partisanship plays a significant role in determining how Americans view mask requirements, these effects are separate from the effects of gender identity; see Figure 4. There is no sign of an interaction effect such that masculinity has a differential effect among Republicans relative to Democrats, for instance.

Instead, the biggest differences between men are tied to gender identity. Among men who identify as “completely masculine,” those who say that their gender identity is “very important” are less likely to support mask requirements than those who say that their gender identity is “not important.” However, men who identify as “slightly” masculine are not significantly less likely to wear a mask if their gender identity isn't as important to them. It should also be noted that this measure is of attitudes toward mask requirements; in comparison with the UAS data, it seems as though there may be some men who oppose mask requirements but report having worn a mask regardless. Still, the numbers are similar enough that this does not seem to be a major concern.

This is in sharp contrast with the results for women. Among women, partisanship matters, with Republican women showing less support for mask requirements, but gender identity has no significant effect at all. For independent men, moving from a gender identity that is “not at all” important to one that is “very important” decreases support for mask requirements by 27 points (81 to 54). Among women, the same move decreases it by just 3 points (from 86 to 83). Among Republican women, the difference is only 4 points.

## CONCLUSION

While partisanship plays a significant role in driving responses to COVID-19, there is enormous variation within partisan groups, largely driven by men who identify at the extreme end of the gender identity spectrum. This effect is exaggerated among men who feel that their gender identity is central to their overall identity.

It is tempting to view the failure to wear a face mask, or reject masking requirements as a problem of men, but it is not about sex: it is about gender, specifically masculinity among men. As has often been noted, masculine gender identities are fragile, and men are often looking for ways to assert their gender identities to both themselves and to those around them. Signaling that they are unconcerned about the prospect of getting sick may be working as a masculinity display for these men—one that potentially endangers those around them.

## Supplementary material

For supplementary material accompanying this paper visit https://doi.org/10.1017/S1743923X20000616. click here to view supplementary material

## References

[ref1] Bem, Sandra Lipsitz. 1981 “Gender Schema Theory: A Cognitive Account of Sex Typing.” Psychological Review 88 (4): 354–64.

[ref2] Bittner, Amanda and Elizabeth Goodyear-Grant. 2017a “Digging Deeper into the Gender Gap: Gender Salience as a Moderating Factor in Political Attitudes.” Canadian Journal of Political Science/Revue canadienne de science politique 50 (2): 559–78.

[ref3] Bittner, Amanda and Elizabeth Goodyear-Grant. 2017b “Sex Isn't Gender: Reforming Concepts and Measurements in the Study of Public Opinion.” Political Behavior 39 (4): 1019–41.

[ref4] Cassino, Dan, and Yasemin Besen-Cassino. 2020 “Political Identity, Gender Identity or Both? The Political Effects of Sexual Orientation and Gender Identity Items in Survey Research.” European Journal of Politics and Gender. Published online June 23. 10.1332/251510820X15912551895078.

[ref5] Connell, Raewyn W. 1995 Masculinities. Berkeley: University of California Press.

[ref6] Courtenay, Will H. 2000 “Constructions of Masculinity and Their Influence on Men's Well-Being: A Theory of Gender and Health.” Social Science & Medicine 50 (10): 1385–1401. 10741575 10.1016/s0277-9536(99)00390-1

[ref7] Gibson, Michelle, and Bernard J. Denner. 2000 Men's Health Report 2000. The MAN Model: Pathways to Men's Health. Daylesford, Victoria, Australia: Centre for Advancement of Men's Health.

[ref8] Mahalik, James R., Shaun M. Burns, and Matthew Syzdek. 2007 “Masculinity and Perceived Normative Health Behaviors as Predictors of Men's Health Behaviors.” Social Science & Medicine 64 (11): 2201–9. 17383784 10.1016/j.socscimed.2007.02.035

[ref9] McDermott, Monika L. 2016 Masculinity, Femininity, and American Political Behavior. New York: Oxford University Press.

[ref10] Sabo, Don. 2013 “Masculinities and Men's Health: Moving toward Post-Superman Era Prevention” In Men's Lives, eds. Michael S. Kimmel and Michael A. Messner. Boston: Pearson, 213–30.

[ref11] Schneider, Monica C., and Angela L. Bos. 2019 “The Application of Social Role Theory to the Study of Gender in Politics.” Political Psychology 40 (1): 173–213.

[ref12] Westbrook, Laurel, and Aliya Saperstein. 2015 “New Categories Are Not Enough: Rethinking the Measurement of Sex and Gender in Social Surveys.” Gender & Society 29 (4): 534–60.

[ref13] World Health Organization (WHO). 2020 “When and How to Use Masks.” https://www.who.int/emergencies/diseases/novel-coronavirus-2019/advice-for-public/when-and-how-to-use-masks (accessed June 19, 2020).

