# Supplementary material for: Of Masks and Men? Gender, Sex, and Protective Measures during COVID-19
Source: Politics & Gender. 2020 Dec;16(4):1052–62. doi: 10.1017/S1743923X20000616 (PMC7853746; doi:10.1017/S1743923X20000616)
Supplement: Supplementary file 1 [file S1743923X20000616sup001.docx]

*Appendix: Full Specification of Models*

Data from the USC-Dornsife Understanding America Survey are used with permission. Their access policy requires the following disclaimer: “The project described in this paper relies on data from surveys administered by the Understanding America Study, which is maintained by the Center for Economic and Social Research (CESR) at the University of Southern California. The content of this paper is solely the responsibility of the authors and does not necessarily represent the official views of USC or UAS.” The data reported in the paper is accurate as of the June 24, 2020 data release. UAS data have been made freely available to interested researchers.

Most of the results are taken from FDU Poll data. Independent variables used in the logit models presented include partisanship, coded as dummy variables for Independent and Republican, with Democrat as the excluded baseline category. These variables are coded as 0 if the respondent is not part of the partisan group, and 1 if they are. 32% of the sample are Democrats, 42% independent and 26% Republican.

The Gender Identity item is as described in the main text. On the six point scale (1-6, with one being completely masculine, and 6 being completely feminine), the overall mean is 3.5, with a standard deviation of 2.0. The mean score for men is 1.7 (sd 0.9), and for women it’s 5.2 (std dev 1.0).

Sex is coded as 1 for self-reported men, and 2 for self-reported women. 49% of the sample is men, and 51% are women

Gender importance is also described in the main text. It is a 4 point scale running between “Extremely Important” (1) to “Not Important at All” (4). Mean score is 1.8, with a standard deviation of 0.9.

Education is coded on a 3-point scale, with 1 being respondents with a High School degree or less (21%), 2 being those with some college or a 2-year degree, but no Bachelor’s degree (28%), and 3 being those with at least a 4-year degree (50%). Mean score on this scale is 2.3, with a standard deviation of 0.8.

Respondents were coded as non-white using a dummy variable (0 for non-Hispanic white; 1 otherwise). 29% of the sample was non-white.

Age was coded on a 4-point scale, with 1 being 18-29 years of age (19%), 2 being 30-49 (34%), 3 being 50-64 (27%) and 4 being 65 or older (20%). Mean score was 2.5, with a standard deviation of 1.0.

The dependent variable, asking about the acceptability of government mask requirements, was coded as a 0-1 dummy, in which 1 means that such a requirement is acceptable, and 0 means that it is not. Respondents who said that they didn’t know, or refused to answer the question (12) were excluded from the analysis. Overall, 80 percent of the sample said such requirements were acceptable.

These variables were used across the three models shown. Model 1, which contains all of the variables and the interactions, has a total N of 934, and a pseudo-R^2^ of 0.18. Model 2, which leaves out the interaction effects, has an N of 934 and a pseudo-R^2^ of 0.16. Model 3, which excludes the Gender Importance variable (both the main effect and the interactions, has an N of 934 and a pseudo-R^2^ of 0.17.

*Appendix: Similarities between single item and other gender identity measurement scales (To Be Included in the Online Appendix)*

The results of the 6-point scale match up nicely with Bittner and Goodyear-Grant’s (2017a, 2017b) 101-point scale. They find similar, though slightly lower, proportions of both men (39 percent) and women (36 percent) identifying with the extreme gender category most associated with their sex: our extreme categories seem to be roughly equivalent to 0-5 and 95-100 on their 101 point scales. We also find about the same proportion of respondents in the gender categories conventionally associated with the opposite sex (8 percent in their sample, 7 percent here, mostly women in both). Given that their data was from an online sample in Canada, and ours is a live-caller telephone poll in the US, the similarities are reassuring.

The results also align well with McDermott’s multi-dimensional BSRI data, which is drawn from a US online sample. In her work, individuals with high levels of femininity are more likely to identify as Democrats, while individuals with high levels of masculinity are more likely to identify as Republicans, with a stronger relationship between femininity and being a Democrat (24 points versus 18 points). In comparison, our data finds that individuals at the highest level of masculinity are 18 points less likely to identify as Democrats, and 18 points less likely to identify as Republicans. While the unidimensional nature of our measure means that we cannot differentiate between McDermott’s “undifferentiated” and “androgynous” categories, the partisan distribution of individuals in these categories is very similar to the distribution of individuals in our middle (“slightly”) categories, with the biggest group of these individuals in the Democratic category, the second most as Independents, and relatively few Republicans.
